# Dementia Care for Europeans in Thailand: A Geography of Futures

**DOI:** 10.1177/00027642221075263

**Published:** 2022-02-28

**Authors:** Geraldine Pratt, Caleb Johnston

**Affiliations:** 1 Department of Geography, 175066 University of British Columbia, Vancouver, BC, Canada; 2 School of Geography, Politics and Sociology, 5994 University of Newcastle upon Tyne, Newcastle upon Tyne, UK

**Keywords:** Dementia care, international medical migration, regime of anticipation, futures, transgender, Karen highlander, Thailand

## Abstract

We explore the creation of private care facilities around Chiang Mai in northern Thailand to provide dementia care for people from the Global North. We draw on three periods of ethnographic observation at care facilities, and interviews with Swiss and British owners and family members, as well as Thai managers and care workers. We locate this offshoring of dementia care from the Global North to South within existing underfunding of dementia care in the Global North and a “regime of anticipation” built around expected substantial growth in the numbers of people living with dementia. These facilities are opening new futures for those who migrate for care as they leverage their relative wealth and privilege to purchase care in Thailand. In line with other readings of international health migration, we note the negative impact of this state-supported privatized industry on the availability of nurses and care aids in public hospitals in Thailand. We then venture into less examined and expected futurities, namely, the opportunities these facilities provide to two groups of stigmatized Thai workers: transgender and Indigenous Karen caregivers.

After a lucrative corporate career in logistics, working in the United Kingdom and across Europe, Peter retired in his 50s to Thailand. In 2007, Peter and his Thai wife purchased a bankrupt hotel outside Chiang Mai. It was around that time that he returned to England to visit his mother, who was residing in a care home managed by one of the country’s largest private social care providers. With four caregivers for 40 residents, he was deeply distressed by the conditions of care. Unable to secure a better option in England, Peter relocated his mother to his resort, and refashioned it as a care facility. His facility now employs around 100 workers, including four full-time registered nurses, two occupational therapists, a massage therapist, and approximately 45 nursing aides. It has a 1:1 carer to patient ratio and a number of “care packages” available depending on level of care required. Of the 92 independent rooms, 13 are dedicated to intensive 24-hour care of people in the advanced stages of dementia, and 66 to residents with less complex care needs. Born from necessity in the present, Peter recognizes the growth potential: “I think we’ve got 30 years of growth in dementia, with quite frightening numbers coming along.”

Peter is part of a growing number of western entrepreneurs who—since 2002—have created and now manage care facilities for aging westerners living with dementia in Thailand. While the exact number of care facilities in Thailand is unknown, Bender and Schweppe (2019) estimate nineteen,^1^ with the majority clustered around Chiang Mai.

This entrepreneurialism is a new twist on racialized global care chains that are rooted in histories of uneven development. The novelty is that those in need of care are the ones who migrate. As Peter’s case indicates, this offshoring of dementia care responds to the present “care crisis” experienced (with variation) across the Global North (Bender et al., 2020). This is a crisis driven by the sustained underfunding of social care, the privatization and financialization of existing long-term care facilities and critical shortages of care workers in many countries (Armstrong & Armstrong, 2019; Harrington et al., 2017; Horton, 2019).

Entrepreneurial activity in Thailand is equally driven by investors speculating on the projected “tsunami” or emergency of dementia. The World Health Organization, for example, projects that by 2050, the number of people aged 80 and over will quadruple to 395 million, with an estimated one in six likely to develop dementia, and that by 2030, 40 million new health and social care jobs will be required to care for increasing numbers of people with dementia (2017). For many countries across the Global North (and beyond), the projected future costs of dementia care are staggering, generating concerns about dementia care absorbing a disproportionate share of government resources (Alzheimer’s Disease International, 2018). The offshoring of dementia care to Thailand is an example of what Adams et al. (2009) have called “regimes of anticipation”: historically and geographically contingent responses to issues in health, medicine, and technoscience that are shaped by speculation and prediction. “Crucially,” they write, “the future increasingly not only defines the present but also creates material trajectories of life that unfold *as anticipated by those speculative processes*” (248).

Geographies are made through actions propelled by anticipation. Places instantiate a “*folding* of futures and pasts into a present where, in turn, futures are constantly being produced. [...A]ny and all geographies are made through this folding and unfolding” (Anderson & Adey, 2012, original emphasis 1530,1533). Dementia care resorts for Europeans in Thailand are places that fold histories of global political economy into the present in anticipation of the future. We begin by situating these facilities within a broader literature on medical, retirement and lifestyle travel/migration, and within histories of uneven relations between Global North and South.

We then venture into a more speculative consideration of unexpected futures, ones that deviate from the more expected storyline of unequal relations between Global North/Global South, to attend to other hierarchies and anticipations. We find these futures in relation to racialized/ethnicized Karen (hill tribe) and transgender people who provide care at these centers. That we find such futures in racialized/ethnicized and trans* workers is especially unexpected because these categories of people tend to be marginalized in traditional (western) accounts of futurity. Smith and Vasudevan note that the “epistemological grammar” of colonialism (i.e., of advanced and developing societies) “centers futurity in the white subject and *disqualifies* non-white subjects from full humanity and thus from a forward-oriented agency, and confines these subjects to zones of death and sacrifice in *service* to white futurity” (2018, 211, original emphasis). So too, it has been argued that futurity is typically heterosexed because reproduction and the child are so dominant within visions of the future. A substantial literature in queer theory is oriented to alternative queered conceptions of non-heterosexed temporalities “to catch a small hold of many futures, to invite futurity even as it refuses to script it” (Puar, 2007, xix; see also Munoz, 2009; Oswin, 2012). We suggest that European-owned dementia facilities in Thailand allow some racialized and transgender care providers to catch hold of a future otherwise unavailable to them.

To develop this analysis, we draw on research carried out between January 2019 and January 2020, when we conducted participant observation and interviews at five facilities that specialize in dementia care for overseas residents. On three separate field trips we lived for 1 week at each of two facilities (that is, 3 weeks in total at each), one Swiss and one British owned, staying on-site in accommodation available to visiting family members. During this time, we did participant observation and conducted unstructured in-depth interviews (typically about an hour in length) with 10 members of management teams (six individuals interviewed twice), as well as 16 nurses, occupational therapists, and care aides, and family members (spouses and adult children) of 13 residents living with dementia (in some cases interviewing family members multiple times). Multiple mediations and diverse power dynamics were at play. The managers knew of and approved the interviews with their employees, and in one facility, managers identified the workers to be interviewed and scheduled the interviews. We relied on a Thai interpreter for interviews with workers. This interpreter was female, from northern Thailand, and a recently graduated undergraduate student who negotiated her own status with interviewees. As researchers from the Global North we were also interpreted, including by managers, who were alert to the possibilities our research affords for marketing and publicity. Although we spent time with persons living with dementia at these centers, we did not interview them because of the challenges of establishing informed consent.

## Uneven Geographies of Care

Private care facilities catering to non-Thai European and North American persons living with dementia exist within longstanding, if ever evolving, uneven geographies of care. In the UK, women are 2.5 times more likely than men to provide intensive 24-hour care to persons living with dementia, 2.3 more likely to be providing this care for more than 5 years, and wives receive less support from friends and family than husbands caring for their spouses in similar circumstances (Alzheimer’s Research UK, 2015). Finding care in Thailand for partners living with dementia gives much-needed relief from providing this care. At the same time, this solution confirms Vora’s observation that people from places in the Global South (too) often service “the reproduction of the biological and affective life of other places” (2015, 7). It fits within broader trends of care, retirement and lifestyle migration wherein individuals from the Global North move to the Global South to manage the effects of austerity politics and the decline of social care and redistributive institutions in their country of origin (Benson & O’Reilly, 2016). Hayes & Pénez-Gañán (2017) term this “geo-arbitrage.” Such migrants, they argue, face “the logic of expulsion” (2017, 118) from their home countries and manage this by arbitraging their advantage (higher incomes, white privilege, cultural capital) by migrating to countries in the Global South. This international medical travel/migration can no longer be “explained away as an escape valve for ‘exceptions’ but rather [has been] discursively reincarnated as a harbinger of social crises where ‘centers and peripheries will not hold'” (Ormond, 2013, 4).

The Thai state has actively courted this travel, and associated short-term and long-term stays, since the late 1990s and Thailand is now a global leader attracting international health travelers (Botterill, 2017; Ormond, 2013). This has had complex impacts on Thai society, including hiving off health resources from the national population. There have been concerns about nursing shortages in Thailand for many years, despite a three-fold increase in the production of trained nurses from 1985 to 2013 (Witthayapipopsakul, 2019). In the 1960s–mid 1970s, a nursing shortage in Thailand was attributed to the “brain drain” of trained nurses (and doctors) migrating to the United States (Meucke & Srisuphan, 1989). Rapid privatization of health services and infrastructure in the late 1980s and 1990s created further nursing shortages in the public sector. As one aspect of this, the Thai government has invested substantially in building private hospitals, primarily in large urban areas, with the aim of creating world-class infrastructure to attract paying clients from around the world (Abhicharttibutra et al., 2017; Cohen, 2008, Kunaviktikul et al., 2015). Intertwined histories turn up in the present. Wilson (2010) traces the genesis of Thailand’s globalized medical tourism industry within a longer geopolitical history, in particular the US investment of military personal and medical training during the IndoChina wars. The infrastructure of medical tourism, she argues, was produced through the intimate public–private partnership of “Thai kin-corporate capital” and the strategic investments of the entrepreneurial state (ibid, 133).

Connell quotes the Secretary General of the Thai Holistic Health Foundation as saying, “In the past we had a brain drain; doctors wanted to work outside the country to make more money. Now they don’t have to leave the country, the brain drain is another part of our own society” (2016, 87). Thais with modest and even mid-level incomes are increasingly dependent on the national health-care coverage scheme, brought in by Thaksin government in 2002. NaRanong and NaRanong make note of the impact on middle-class Thais who can no longer afford the services provided by private hospitals “on which they used to rely on a regular basis.[...] Moreover, since hospitals for medical tourists have lured many highly skilled physicians and specialists out of public and teaching hospitals, the majority of Thais will henceforth probably receive health-care services of lesser quality” (2011, 341; see also de Arellano, 2007).

As with other forms of medical travel, dementia centers catering to overseas clients place strain on an already stressed public care infrastructure in Thailand’s aging society. Most of the nurses and occupational therapists that we interviewed described their movement from public and private hospitals into this privatized form of dementia care. Take Aom^2^ as one example. Originally from Lamphun province, she graduated from the Faculty of Nursing at Payap University before working for 8 years in the Cardiac Care Unit in Chiang Mai’s McCormick hospital. Looking for a change from the hospital’s stressful working conditions, she took a job at a European-owned care facility as one of its four registered nurses in 2019. Alternatively, Chariya, graduated from Chiang Mai University as a registered nurse, after which she worked for 5 years in a local government hospital before landing a job in elder care. “I wanted to work in a different field […] that is not a hospital,” she explained. “Working here makes me live my life a little slower. […] I have time to think, to consider each case more. And I feel happier.” Medical staff interviewed had collectively moved from public to private care and their decisions were motivated by the desire to escape difficult and often stressful working conditions, the possibility of higher salaries, and in some cases, the opportunity to practice a different and slower pace of care work.

When queried on the possibility of simultaneously benefiting from lower wages in Thailand and placing a drain on local resources, one owner responded sympathetically: “Yes, that definitely sounds like exploitation, but it is not like that at all.” “We employ personnel, we are building institutions, and we are a good employer,” he elaborated. “And we generally pay wages that are 20% above market value, simply because I do not want any fluctuation. I do not want to constantly confront our guests with new faces and people, etc. I want to have the best and pay them more and establish a certain stability that way.” He insisted: “We are not stealing any gold treasures or diamonds or natural resources to bring them to Switzerland and make profits there. On the contrary.” His defence nonetheless makes the case that he is benefiting from uneven geographies of the global economy. His capacity to pay 20% above the market rate in Thailand rests on the discrepancy of the costs of care in Thailand as compared to Switzerland. Not the theft of diamonds to be sure, but the siphoning of commodified labor, that is, trained health workers, to address the needs of Europeans.

One challenge of maintaining a stable workforce in a growing business has been ensuring a steady stream of nursing aides, who represent these facilities’ largest work force and are a key to providing (and marketing) their 1:1 care ratio. Two of the largest facilities ensure this stable labor supply through close links with local nursing-aide schools in Chiang Mai. These offer one-year training programs, which include 2 months of hands-on work experience. “There are four nurse aide training schools,” one owner explained, “So, we hit them. We’re trying to get them when they come out. We have a lot on “work experience,” which is really good. [When] you have people on “work experience,” especially if you have them for more than a month, you know whether they’re good or not. And if they’re good, we’ll offer them the job.” But “half of them aren’t going to make it,” he qualified. “We [also] put adverts in [the newspaper]. We put [signs] on the side of the road. It’s getting tougher, because there’s more places like this. I mean, as the years go on, Thailand will have big employment problems.” Unacknowledged is the fact that his facility and others like his are themselves placing stress on the supply of nursing aides.

These dementia care centers and transnational care-seeking migrants to Thailand are thus a new twist on an old story of “brain drain” and transnational care chains. Researchers of retirement and lifestyle migration have complicated the story by exploring the complexity of privilege, precarity, and vulnerability among those who migrate (Botterill, 2017; Lafferty & Maher, 2020). We detect other complexities and focus on other futures emerging within the outsourcing of dementia care in relation to care workers. We draw attention to other futures for individuals who are otherwise at the bottom of racial and ethnic formations and gender orders in Thailand. The narratives that follow are singular but suggestive. The migration of persons living with dementia for care not only alters the lives (and significantly improves the care) of those who migrate, but also impacts the lives of those who provide the care. Let us be clear: hierarchies of race and gender in Thailand are deeply embedded in histories of unequal global economies and relations of semi-coloniality, and we are not celebrating the opportunities forged by forward-looking westerners. Rather, we are suggesting that the folding and unfolding of geographies across Global North and Global South, which occurs within these facilities, can create futures unanticipated in either Thailand or Europe.

## Unsettling Racial Exclusion

Asked whether he employed Karen workers, a British care resort owner put it bluntly: “I’m biased about hill tribes.” He employed two highlander workers as carers but explained his reluctance to hire more: “First of all, the old [previous] queen [of Thailand] got interested in hill tribes. And then the missionaries got interested in hill tribes. They’ve had a lot of money pushed on them. They’ve...we’ve grown a culture of dependency in the hill tribes.”

This British owner’s articulation of hillside tribes as a distinctive and dependent population is fitting, given that the European idea of racial classification is credited with the differentiation of Thai from non-Thai hill tribes (Laungaramsri, 2004; Streckfuss, 1993). The racialized distinction emerged at the end of the 19th century, when it was adopted by Bangkok elites as part of the process of nation building (Thongchai, 1994). Within this classificatory schema, the Karen, along with eight other highlander groups, were classified as *khon pa*, that is, as uncivilized natives, and as members of a non-Thai ethnic category that stood apart from the new Thai citizen subject, locked in the premodern past. “While racial homogeneity served as a form of resistance against European colonialism,” notes Laungaramsri, “racial differences between the Thai and the non-Thai who lived in the marginal realm provided a basis for a reference point for the historical evolution of Thai civilization” (2004, 27). Renard (2004) argues that the category Karen is itself a modern construction that joins an otherwise culturally diverse group who share little more than a common language. He views it to be a product of Christian missionaries, colonial and postcolonial ethnographic research and Thai nation building. The official designation of “hill tribe” (*chao khao*) in 1959 constituted highlanders as outsiders (non-Thai) living within the territory of the Thai nation. As Laungaramsri notes: “The term *chao khao* has a double meaning. Its literal meaning is “hill” or “mountain people.” However, when placed in a punning manner with the term *chao rao* (“us people”), the term acts as a binary opposition in its other meaning of ‘them people” (2004, 29).

Such histories of racial classification and nation building frame exclusion in contemporary Thailand—a country with one of the largest stateless populations in the world (Flaim, 2017; Cheva-Isarakul, 2019). It is estimated that approximately 40% of those designated as hill tribe are stateless and are thus excluded from formal Thai citizenship. The Karen historically have lived closer to lowland Thais, have been more integrated in lowland life and have higher rates of citizenship. But still, stereotypes have taken hold. Hill tribes in general are often associated with illegal opium production, the destruction of forests and watersheds (from swidden agricultural practices), premodern (and non-Buddhist) animistic beliefs, and political insurrection. Among designated hill tribes, the stereotypes of Karen tend to be somewhat more benign: they are imagined to be non-ambitious, poor, destitute, shy, clumsy, peace loving, and subservient (Laungaramsri, 2004). Nonetheless, even these stereotypes hardly speak to the image of an ideal employee.

Karen workers were employed at all of the European care facilities we researched. Not all employers articulated the common stereotypes and several actively disregarded them. In one Swiss-owned facility, the Swiss nursing manager had identified Nan as a possible replacement for herself. Nan is Karen from Lamphun: “I am from the Karen tribe and very proud of it”^3^ She studied English at university in the Faculty of Education, and originally sought a job to take care of foreigners to practice her English. “I wanted to take a job,” she told us, “because I could spend time with a foreigner and practice my language skill [...] which would make me into this version of Nan [...]” She was well aware of the stigmatization of Karen people:

About coming from a Karen tribe, I have always heard that it is not equal between Thai people and Karen people. No one ever tells me directly it’s a 100% fact. It’s more just a feeling that I feel it’s not equal. So, I have to make my weak spot become a highlight [so that I can] stand in the same spot as others. Which can be proved by work and success. This topic is the thing that I always hear about since I was a child. And I have to [work harder] to be in the same position as they are. When I was studying in university, most of my classmates’ parents are teachers in the high ranked school in Chiang Mai. But my parents are only Karen farmers. So, I have to do everything to make myself shine, to prove that Karen can also be successful.

She noted that two or three other Karen women worked as care aides at the facility: “When I have to teach these people, I always teach them extra because I don’t want them getting looked down upon.”

Despite having no training in nursing or as a care aide, Nan has been identified as the replacement for the current nurse manager (the most senior management care position) because, we were told, she is so excellent at her job. Her job has expanded significantly since she came to work at the facility, at first only on a part-time basis. The Swiss nurse manager positioned Karen workers as inhabiting a space between Thai and western cultures, particularly in a level of ease with and capacity to care for people in the process of dying.^4^ Nan’s employers have supported her to get the necessary training to assume a leadership role. In the meantime, she has been identified as especially gifted in palliative care, and as the favored employee to go to the airport to receive arriving families and care residents and to do the difficult work of settling new residents into the facility. The nurse manager is exploring securing a passport for Nan so she can accompany her on recruiting trips to Europe. Her supervisors recognized the resentment Nan’s growing authority created among the Thai nursing staff: the “original Chiang Mai people don’t always go well with the tribal people. They say they’re superior.” But they feel that it has lessened (and will dispel) over time.

Our sample of one is clearly just suggestive. It does not allow a comparison across British and Swiss owners, who may think about and act upon racial classification differently. Nan may be the exception to the rule. (Although to be clear, Karen workers were employed at other facilities.) Or it may be that Swiss and/or other non-Thai employers, are less embedded within hierarchies and temporalities (i.e., modern/premodern) of racial classification in Thailand and less likely to operate within them. It is likely that Europeans receiving care do not detect Karen as a distinctive ethnic group. We are not suggesting these employers or those receiving care are post-racial in their thinking but that there is a history and geography to racial stereotyping. Likely fully imbricated in Western racial thinking, classificatory systems in place in Thailand are less familiar to (at least some) European managers and care recipients and hold less meaning for them. This may create some openings within otherwise foreclosed futures for Karen people.

## Unsettling Sexual Exclusion

One owner recalled an email received by the Thai management company (which he was then paying to run his resort for him), demanding a strict dress code for his transgender workers.^5^ “When we were managed by [company name],” he recounted, “[they] sent me a message which said: ‘The boss has decided that if you employ ladyboys,^6^ they must dress as a man.’” The British owner pushed back: “I said, ‘It’s not going to happen in my resort.’… cause that’s practically saying, ‘You can’t work for me’ [...] You’re basically saying, you can’t be a ladyboy while you work for us.” The directive from the Thai management company might seem surprising^7^ given Bangkok’s status as one of the first “gay capitals” in Asia (Jackson, 2009) and as a world leader in the field of sex reassignment surgery (Aizura, 2018; Ocha, 2013). Homosexual behavior in Thailand does not necessitate all-encompassing identification or necessarily constitute a transgression of sexual and gender norms (Sinnott, 2004). Western categories, Sinnott observes, “snag [...] and buckle [...] when forced into translation” in the Thai context (2004, 2). Even so, Thailand has longstanding and substantive transgender communities.

This noted, access to gender affirmation procedures repeats the dynamic of dementia centers: it is largely available to wealthy clients from the Global North (Aizura, 2018). Further, everyday attitudes toward transgender persons within Thai society have been described in ambivalent terms as “tolerant but unaccepting though not homophobic” (Ocha, 2013, 85). Sinnott details how stigmatizing discourses of sex/gender deviance have been disseminated by state-based Thai educational institutions and through print media. “State officials, the print media, and academics,” suggests Sinnott, typically link “‘homosexuality’ to problems in national development, national image, or imputed Thai traditional morality and culture” (2004, 183). Homosexuality, Sinnott argues, has become a “discursive device” to discuss the negative impacts of westernization on Thai culture (188). As Käng argues, “kathoeyness”, despite long histories rooted in Thai history, culture and public life, often produce deep “social anxiety” and are typically viewed as “detrimental to national identity and [biological] reproduction” (2012, 476). In the late 1990s anxiety about gender nonconformity was expressed through a number of high-profile (and contested) state condemnations: for example, in 1998 the Public Relations Department announced the restriction of images of transgender persons in television programs; in 1996–7 the system of teachers’ colleges announced a ban on homosexual students (Sinnott, 2004). Since the 2006 coup, popular representations of gender nonconformity had tended to be more sympathetic but Kang (2012) ventures that these have nurtured a counter discourse of concern about the contagion of homosexuality. In “nationalist discourse expansion of kathoeyness is linked to a pathologising discourse, expressed as a loss of Thainess and the inability of Thai society to reproduce itself” (481).

So too, increased civic tolerance need not displace employer discrimination. Many transgender persons still find work in the entertainment and commercial sex industries (Ocha, 2011; 2013, 2015). *Sao praphet song* ^8^ are often edged out of mainstream jobs gendered as male and into stereotypically female jobs. We perhaps should not have been so surprised, then, that a good number of workers at European-owned care facilities are transgender. The British owner quoted above was highly alert to the fact that care work offers a potentially valuable labor market for transgender women: “They tend to work in hospitals and at 7–11 [convenience stores]. [They’re] in care because they’ve done the nurse aide training course.” “[But] I’ll rephrase that,” he qualified, “The lower-class hospitals, the government hospitals and the cheap hospitals hire them. The expensive international ones, don’t. […] There’s a massive, huge glass ceiling against ladyboys.” In his estimation, given their higher wages than government hospitals and the possibility of more tolerant views on transgender sexuality, European-owned care facilities in the Chiang Mai open important employment opportunities for transgender care workers. This is in line with finding that Thai workers (cisgender and trans*) regard their work in gender reassignment clinics (which serve mostly white transgender persons from the Global North) as higher status than other forms of care work because of higher wages, along with the opportunity to learn English and other skills.

The British owner of the care facility quoted above estimated that up to 50% of his workforce was either transgender or in a gender nonconforming relationship. “I said [to] my manager, I reckon 40% [of the facilities’ workers] are gay. And she said, ‘I think you’re underestimating.” He drew particular attention to hiring a transgender person, who we will call Luna, for a senior supervisory role: “I interviewed this girl for a supervisor’s job,” he explained. “She said, ‘Are you happy to take me on?’ I said, ‘Why do you even ask the question?’” The question was asked because the worker—at the time of her interview—had been compelled to provide her Thai ID card (which includes one’s biological sex). As the employer explained: “They can’t hide it. Every single employer gets a copy of the ID card.”

Hiring Luna in a supervisory capacity *is* worthy of note. Luna had moved away from Chiang Rai to Bangkok in her early twenties. Her family had said they would not support her and Luna worked hard to put herself through a two-year technical nurse course by working in a call center and singing and emceeing as an entertainer. Her first care job was at a Swiss-owned dementia care facility for Europeans located in Phuket, where she worked for 6 years before returning to northern Thailand to work for the British facility owner quoted above. Attaining a job that involved supervising staff of approximately 40 caregivers was a significant accomplishment.

We heard also from those who received care about their tolerance and appreciation of transgender persons as caregivers. From a British male care recipient in his late 80s, who raised the issue himself^9^:

Thailand is, is a country, at least as far as I’ve seen, where things.... which are peculiar in the West, certainly in England at the moment, [where] people can’t work out whether it’s right to accept people of the same sex falling in love, marrying, having children and so on. Here, if you want to be, if you’re born male and you want to be a woman, then you behave like a woman, you dress like a woman, you are a woman. We have several of them here, and a lot of them, they -- you would have no idea, I mean one of the most beautiful women I’ve met here is actually a ladyboy. And you [referring to interviewers] all know her as well, actually. But you don’t know which one [laughs]. But it’s, it’s lovely. [...] It’s lovely to be surrounded by such people. I mean every Friday I have a massage by two ladyboys, normally. But to me they, you know, they’re just normal people and they treat each other normally. I do think it’s rather lovely.

It is difficult to know what to make of this statement. Is this an elderly British man restating a western erotic imaginary of Thailand as bordello (McKinnon, 2005)? Or something more interesting and nuanced? Far from his own culture, physically vulnerable, highly dependent for care for himself and his wife who has advanced dementia, his pleasure in engaging transgender people as people, simply as normal people, seemed genuine. In on the secret, coming to know himself and the world as more tolerant of difference, perhaps it *is* rather lovely. It is hard to say. It is clear that he has reconfigured transgender as a source of valued care. This, as Aizura notes, “flies in the face of feminist scholarship on reproductive and affective labor that has focused on cisgender women as subjects of such labor and opens a space to inquire about transnational queer or trans chains of care” (2018, 196). Such an inquiry can both deepen a critique of global power structures across Global North and South, and open expected lines of solidarity, including (possibly) unlikely solidarities among those rendered disposable (in very different ways) through the global privatization of health and social care.

It would be a naive, however, to stabilize this reading and a mistake to overstate European tolerance of sexual diversity. Tolerance of sexual diversity is taken in many countries in the Global North as a measure of progressive western liberalism (and the implicit backwardness of places where such tolerance is not exhibited) and thus replays an old story of western enlightenment and superiority (Butler, 2008; Puar, 2007). The embrace of sexual diversity for economic advantage can stop well short of full inclusion (Oswin, 2012). Luna was fired by the British owner quoted above for his (in our understanding) misapprehension that she had been leading guests and staff to another facility. “But then she was disloyal,” he told us, “so I got rid of her.” Asked what she was now doing, he said that she was working nearby as a ladyboy in a beauty salon. In fact, Luna had returned to her former employment as a trained, skilled, and experienced practical nurse in Phuket. Stereotyping and discrimination sit very close to the surface. The European care sector may nonetheless offer small openings, no doubt complicated by inequalities, stigma, and distrust, toward different futures, less possible in other sectors of the health-care sector.

## Futures of Care

Meeting with Professor Dr. Kannika, 80 years old, a semi-retired Thai oncological researcher and a force behind the promotion of Chiang Mai as a long stay city through the Chiang Mai Health Services Promotions Association, she mused about her relationship to the future: “To be Buddhist, we have to think about what is in front of [us], not the past. Look a little bit forward, but not so far.” Focused on the near future then, she believes that Thai people will have to learn new ways to care for the aging population: “Right now the situation is changing. We have to work. We have to earn some money [making it more difficult for families to provide care.] So what happens? I don’t know.” Of the European dementia centers in Chiang Mai, she had this to say: “at least they create jobs. And we can learn from them. We learn from each other. I don’t mind: you come or you go. At least we can get the good part. And then [laughing] we convert.” Dr. Kannika is putting the European dementia centers in their place, among other health traditions that are of interest to her: for instance, Chinese, Ayurvedic. She tells us of interesting models in rural areas she has visited in Chiang Mai province. She is envisioning new relations of care through the folding (and unfolding) of models of care from different places.

We have considered a number of geographies of care within a regime of anticipation. Persons with dementia move (or are moved) to Thailand for dementia care to create a future for those who are unburdened of care and a more comfortable shorter term one for those who receive it. European owners make capital investments anticipating a growing market for this care. Swiss and British owned dementia centers in Thailand are established in anticipation that dementia will increase, and that the cost of care will continue to be unsustainable in the Global North. The folding of European families and investment into the Thai health infrastructure, we have argued, replays and thickens some old histories of global relations of power and privilege. The facilities thrive because of wage differentials between Thailand and various countries in the Global North. They draw caregivers away from the public health sector, upon which more and more Thai nationals depend, in the context of an aging society requiring more and more care. Within this, we have argued that new futures are beginning to emerge for a small number of Karen and transgender care providers, who are finding new futures for themselves in senior level jobs within and beyond the folded and unfolded geographies of these European-owned dementia facilities. In this they do more than replay the epistemological grammar of colonialism and white supremacy, and suggest the possibilities for something new, something not yet anticipated in the intertwined regimes of anticipation emerging across Global North and South.

## Notes

1. This does not include facilities catering to Japanese retirees (see Toyota, 2006, Toyota & Xiang, 2012). Given our language skills, methodology of participant observation and the challenge of recruiting family members for interviews, we focused our efforts on facilities where English is spoken.

2. All names are pseudonyms.

3. Interviews at the site of employment are always fraught and in this case employees knew that the interviews had been scheduled with the assistance of a nurse manager. This worker brought forward the topic of her Karen identity; it was not something that we were alert to going into the research, and indeed is still not perceptible to us. Once drawn to our attention we did pursue it with interest when other Karen workers in other facilities raised the subject of their Karen heritage.

4. Part of the construction of Highlanders as marginal is tied to the assumption that they are not Buddhist, and thus their beliefs sit outside the national religion. This is only partially true for the Karen people in Thailand, among whom the conversion to Buddhism has been significant. Although American Baptist missionaries were very active among the Karen (especially in Burma), a survey in 1998 by the Hilltribe Welfare and Development Center of Thailand estimated that 55% of more than 300,000 Thai Karen identified as Buddhist, 17% as Buddhist and animist, and 18% as Christian (Keyes, 2004, 216).

5. While used in reference to all non-normative gender categories in Thailand, *kathoey* is used to describe male-to-female transgender women in Thailand and *tom-dee* for female transgender cultures (see Jackson, 2000; Kang, 2012; Sinnott, 2004; Ocha, 2015; Ocha & Earth, 2013).

6. The term ladyboy typically refers to transgender people who are cabaret performers, beauty pageant contestants and bar-based sex workers, and is a derogatory term.

7. It has been suggested by a reviewer that the management may have been anticipating the owner’s attitudes toward transgendered workers. This may be the case and we cannot adjudicate this interpretation.

8. Although *kathoey* is used in the scholarly literature that we draw on here, *Sao praphet song* (roughly translated as a second kind of woman) is considered to be more respectful than *kathoey*, and certainly ladyboy (Aizura, 2018).

9. We did not raise this issue directly with family members and cannot judge how widely this viewpoint was held among residents.
